# Multidisciplinary, evidence-based consensus guidelines for human papillomavirus (HPV) vaccination in high-risk populations, Spain, 2016

**DOI:** 10.2807/1560-7917.ES.2019.24.7.1700857

**Published:** 2019-02-14

**Authors:** Xavier Martínez-Gómez, Adrian Curran, Magda Campins, Laia Alemany, José Ángel Rodrigo-Pendás, Natalia Borruel, Xavier Castellsagué, Cristina Díaz-de-Heredia, Fernando A Moraga-Llop, Marta del Pino, Aureli Torné

**Affiliations:** 1 Servei de Medicina Preventiva i Epidemiologia, Hospital Universitari Vall d’Hebron – Universitat Autònoma de Barcelona, Barcelona, España; 2 Servei de Malalties Infeccioses, Hospital Universitari Vall d’Hebron – Universitat Autònoma de Barcelona, Barcelona, España; 3 Programa de Recerca en Epidemiologia del Càncer, Institut Català d’Oncologia – IDIBELL CIBER Epidemiología y Salud Pública, Barcelona, España; 4 Unitat d'Atenció Crohn-Colitis, Servei d’Aparell Digestiu; Hospital Universitari Vall d’Hebron – Universitat Autònoma de Barcelona, Barcelona, España; 5 Servei d’Oncologia i Hematologia Pediàtrica, Hospital Universitari Vall d’Hebron - Universitat Autònoma de Barcelona, Barcelona, España; 6 Asociación Española de Vacunología, Barcelona, España; 7 Unidad de Ginecología Oncológica, Instituto Clínico de Ginecología y Obstetricia y Neonatología (ICGON), Hospital Clínic de Barcelona, Barcelona, España; 8 Instituto de Investigaciones Biomédicas August Pi i Sunyer (IDIBAPS), Facultad de Medicina, Universidad de Barcelona, Barcelona, España

**Keywords:** evidence-based medicine, human papillomavirus infection, HPV, vaccines, immunisation, high-risk populations, HIV infection, HIV, men who have sex with men, MSM

## Abstract

Introduction: Although human papillomavirus (HPV) routine vaccination programmes have been implemented around the world and recommendations have been expanded to include other high-risk individuals, current recommendations often differ between countries in Europe, as well as worldwide.

Aim: To find and summarise the best available evidence of HPV vaccination in high-risk patients aiding clinicians and public health workers in the day-to-day vaccine decisions relating to HPV in Spain.

Methods: We conducted a systematic review of the immunogenicity, safety and efficacy/effectiveness of HPV vaccination in high-risk populations between January 2006 and June 2016. HPV vaccination recommendations were established with levels of evidence according to the Grading of Recommendations Assessment, Development and Evaluation (GRADE) system.

Results: A strong recommendation about HPV vaccination was made in the following groups: HIV infected patients aged 9–26 years; men who have sex with men aged 9–26 years; women with precancerous cervical lesions; patients with congenital bone marrow failure syndrome; women who have received a solid organ transplant or hematopoietic stem cell transplantation aged 9–26 years; and patients diagnosed with recurrent respiratory papillomatosis.

Conclusions: Data concerning non-routine HPV vaccination in populations with a high risk of HPV infection and associated lesions were scarce. We have developed a document to evaluate and establish evidence-based guidelines on HPV vaccination in high-risk populations in Spain, based on best available scientific evidence.

## Introduction

Human papillomavirus (HPV) is the main cause of uterine cervical cancer (UCC) and its precursor lesions [1,2]. HPV can also be found in cancerous and precancerous lesions of the vulva and vagina [3,4], penis [5] anus [6], oropharyngeal cancer [7], anogenital warts [8] and recurrent respiratory papillomatosis (RRP) [9]. Nearly 90% of all female genital HPV infections are transient and resolve on their own in the next 2 years [10]. Persistence of HPV happens in 10% of infected healthy women and 1% of them will develop neoplastic HPV-related lesions. In men, duration and persistence of HPV infections are shorter than in women [11].

The estimated impact of newly diagnosed cases of HPV-related disease is high around the world: annually there are 30.9 million cases of cervical precancerous lesions, 32 million cases of genital condylomata and 630,000 cases of cancer (cervix, vagina, vulva, anus, penis, oropharyngeal, oral cavity and larynx) diagnosed [12,13].

HPV vaccination is an effective primary intervention to prevent HPV infection and its associated disease burden. Vaccination is used as part of a coordinated strategy with quality screening programmes and treatment services in cervical cancer prevention [14]. Three HPV vaccines containing virus-like particles (VLPs) are currently available: the bivalent vaccine (VLPs of high-risk HPV genotypes 16 and 18) [15]; the quadrivalent vaccine (VLPs of genotypes 6, 11, 16 and 18) [16]; and the nonavalent vaccine (VLPs of genotypes 6, 11, 16, 18, 31, 33, 45, 52 and 58) [17].

In the first decade of HPV vaccine use, the main strategy worldwide was routine vaccination programmes for adolescent and young women. In Europe, according European Centre for Disease Control (ECDC) information reviewed in 2018, routine vaccination is included in the national immunisation schedule in the majority of countries (n = 29, 93.5%) [18]. In some countries, vaccine recommendations were later extended to include specific high-risk groups as the United States (US) where three doses of HPV vaccine are recommended for: (i) females and males aged 9–26 years with primary or secondary immunocompromising conditions that may reduce cell-mediated or humoral immunity, (ii) men who have sex with other men (MSM), (iii) transgender persons aged 9–26 years, (iv) individuals not adequately vaccinated and, (v) children with a history of sexual abuse [19,20]. In Australia MSM and immunocompromised individuals are included [21] and in Canada individuals with abnormal cervical cytology or a history of UCC and/or genital warts, MSM and immunocompromised individuals [22] are included too. Currently, no global European recommendations for specific groups are mentioned [18] but some countries have begun to develop programmes [23].

In clinical practice, there is growing interest in expanding vaccine recommendations to patients at high-risk of HPV infection and development of related neoplasms. Currently, information on HPV vaccination for these high-risk populations is scarce and recommendations for selective vaccination differ between countries in Europe. Several reasons may explain the lack of guidelines for vaccination in high-risk populations, including: heterogeneity in the definition e.g. age, sex, disease characteristics and variations in the risk of infection, persistence or malignancy; limited published clinical trial data (due to difficulties in conducting appropriately powered studies on vaccine immunogenicity, safety and efficacy in individuals with low-incidence diseases); and limitations associated with establishing recommendations for patient groups not included in the product label. Some of these issues can be viewed in the Supplementary material.

A guideline on HPV vaccination in high-risk individuals, based on the review of current evidence by an independent and multidisciplinary expert panel, was recently released by the Spanish Association of Cervical Pathology and Colposcopy [24]. This paper summarises this evidence-base and process and depicts the guideline.

## Methods

In October 2015, a steering committee was formed in Spain to compile this guideline after a rigorous review of the HPV-associated disease burden and a systematic review of studies investigating the immunogenicity, safety and efficacy of HPV vaccination in high-risk populations. This committee consisted of 10 Spanish professionals including gynaecologists, epidemiologists, paediatricians, infectologists, haematologists, gastroenterologists and preventive medicine specialists with recognised expertise in the area.

### Selection criteria

This review included both randomised controlled trials (RCT) and observational studies (e.g. cohort studies, case reports) that assessed the effects of HPV vaccination on high-risk groups.

Based on clinical expertise of the steering committee and published literature, the following groups were defined as individuals at high-risk of HPV infection and HPV-related disease and were included in the guideline on HPV vaccination in high-risk individuals (Box).

Box **Individuals at high-risk of human papillomavirus infection and related diseases, Spain, January 2006–June 2016**

• HIV infected patients

• MSM

• Inflammatory bowel disease patients

• Women with precancerous cervical lesions

• Patients with congenital bone marrow failure syndrome, primary immunodeficiency or survivors of childhood neoplasia

• Solid organ or haematopoietic stem cell transplant recipients

• Patients with immunosuppressive or biological treatment

• Patients with recurrent respiratory papillomatosis

HIV: Human immunodeficiency virus; HPV: human papillomavirus; MSM: men who have sex with men.

The interventions of interest were the bivalent (Cervarix), quadrivalent (Gardasil) and nonavalent (Gardasil 9) HPV vaccines. The outcomes of interest were the efficacy, effectiveness, immunogenicity and safety of HPV vaccines.

Studies that did not report original data e.g. editorials or reviews were excluded.

### Search strategy

An electronic search was carried out using PubMed to find studies published in English from January 2006 to June 2016. The search terms were a combination of the names and synonyms of the different risk groups, the HPV vaccines and the outcomes of interest. The details of the search strategies are presented in the Supplementary material.

All authors conducted unstructured electronic searches and reviewed their personal files for relevant published papers.

### Selection process

Two authors (XMG and JARP) independently screened the titles and abstracts of the articles identified by the PubMed search and selected articles for full-text reading in accordance with the selection criteria. Full texts for the relevant references were independently evaluated by XMG and JARP to determine their inclusion. Reference lists of included articles were screened to consider further potentially eligible studies (Figure). Any discrepancies in study selection were resolved by discussion between the reviewers until a consensus was reached.

**Figure:**
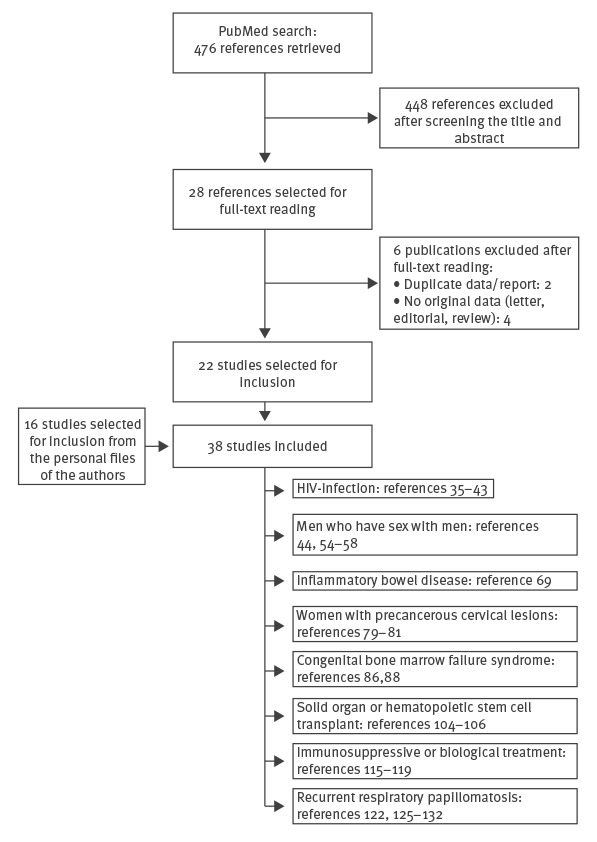
Flowchart of literature selection for review of human papillomavirus vaccination in high-risk populations, Spain, January 2006–June 2016

HIV: Human immunodeficiency virus.

The agreement between reviewers in the selection process was computed using Stata/SE 14.2 for Windows (StataCorp LLC, College Station, Texas) and resulted in agreement of qualitative measures (Cohen’s Kappa = 0.98) [25].

Panel subgroups reviewed the initial literature search, selected references, evaluated evidence, drafted recommendations and summarised the evidence for each section. The content of the guideline was decided by the consensus panel; the evidence evaluation process involved a systematic weighting of the quality of evidence and the grade of recommendation using the Infectious Diseases Society of America (IDSA) version of the Grading of Recommendations Assessment, Development and Evaluation (GRADE) system [26,27].

The developed guideline for Spain was reviewed and endorsed by several groups acknowledged at the end of the article.

## Results

### Human immunodeficiency virus (HIV) infection

The prevalence of HPV infection in individuals with HIV infection is high [28-30]. Within this population, the number of sexual partners increases the risk of infection and reinfection for multiple HPV genotypes and decreases the probability of HPV clearance [29]. Compared with the general population, the persistence of HPV in people living with HIV significantly increases the risk of HPV-related cancers and condylomata.

Three risk subgroups can be identified in the overall HIV-infected population: HIV-infected women, HIV-infected men who have sex exclusively with women (MSW) and HIV-infected MSM. While UCC screening programmes and the improvement in HIV disease control have led to a decrease in the incidence of UCC in HIV-infected women, the incidence of anal cancer in this population is 30 per 100,000 person-years (US, 1996–2007) [31], and the prevalence of condylomata is 5–7% vs 1% in the general population (US, 1991–1998) [32,33]. In line with this, HIV-infected MSW in the US during 1996–2007 have an incidence of anal cancer that was significantly higher compared with men without HIV infection (46 vs two/100,000 person-years) [31].The prevalence of condylomata in HIV-infected MSW in Spain from 2005 to 2009 was 15% [34]. The incidence of anal cancer in the US during 1996–2007 is especially high in MSM with HIV (131/100,000 person-years; rate ratio = 80.3 compared with men without HIV) [31], and the prevalence of condylomata in HIV-infected MSM in Spain from 2005 to 2009 was 28% [34].

#### Rationale for recommendations

Nine papers of HPV vaccination in HIV population were selected [35-43]. Available data on the immunogenicity and safety of HPV vaccines in people with HIV infection support its use for the prevention of HPV-related cancers and genital warts; however, there are limited data on the efficacy of the vaccine (Supplementary material Table S1).

The efficacy results obtained in RCT of MSM [44] cannot be generalised to the HIV population, since the characteristics of the included individuals (age 16–26 years; ≤ 5 lifetime sexual partners; no history of sexually transmitted infections) differ from the majority of MSM with HIV seen in clinical practice. Prospective, RCT evaluating the efficacy of HPV vaccines in the prevention of recurrence in people with HIV receiving treatment for condylomata or premalignant lesions are ongoing [45-47].

Ideally, people with HIV who receive a HPV vaccine should be on antiretroviral treatment and have good immune-virological control at the time of vaccination.

#### Summary of previous guidelines or consensus recommendations

The European Acquired Immune Deficiency Syndrome (AIDS) Clinical Society guidelines recommend HPV vaccination in all individuals with HIV; however, they state that in situations where there is an established HPV infection, vaccine efficacy is questionable [48]. The US guidelines recommend vaccination for HIV females and males through age 26 years [19,20,49].

The 2013 IDSA clinical practice guideline for vaccination of the immunocompromised host, recommends the administration of three-dose of quadrivalent HPV (qHPV) vaccine for females and males with HIV aged 11–26 years [27].

#### Recommendations in HIV patients

HPV vaccination is recommended in both male and female individuals with HIV from 9–26 years (quality of evidence: moderate; recommendation: strong). HIV-positive people aged 26 years and older could also benefit from HPV vaccination (quality of evidence low; recommendation: weak). The use of any of the available vaccines is indicated, although the quadrivalent or nonavalent vaccines are preferred given the high prevalence of anogenital warts in these patients.

### Men who have sex with men

MSM have a high risk of HPV infection and HPV-related disease than MSW [50], with the incidence of anal neoplasia being nearly 20 times higher in MSM [51]. The annual recurrence rate of high-grade anal lesions following treatment may be up to 50% [52]. Data from the US suggest that MSM have a higher rate of condyloma than women do, but a lower rate than MSW [53].

#### Rationale for recommendations

Six papers of HPV vaccination in MSM population were selected (Supplementary material Table S2). One study evaluated the immunogenicity, efficacy and safety of the qHPV vaccine against HPV in healthy males aged 16–26 years [54]. Of the 4,065 men enrolled in the study, 602 were MSM. Antibody GMTs were lower in MSM vs MSW [55]. In MSM, the efficacy of the vaccine for prevention of persistent genital infection by any of the genotypes in the vaccine was 43.6%, while the efficacy for the prevention of persistent anal infection by HPV 6, 11, 16 or 18 was 94.9% [44,54]. The vaccine was effective in preventing 77.5% of the high-grade squamous intraepithelial lesions (HSIL)/anal intraepithelial neoplasia (AIN) secondary to HPV 6, 11, 16 or 18, and 70.2% of the genital warts [44].

The qHPV vaccine has also been evaluated in MSM patients aged 26 years or older. It was effective in the prevention of anal condyloma in a study of 313 MSM without story of anal condyloma or with history of previous HPV-related anal condyloma who had been recurrence-free for at least 12 months (hazard ratio = 0.45; p < 0.05) [56]; it also reduced the incidence of HSIL (AIN) in another study of 202 MSM aged 18 years and older (83.7% of them aged over 30 years) who were treated for histologically confirmed HSIL (AIN) [57]. The recurrence rate of HSIL (AIN) in vaccinated MSM vs those not vaccinated was 10.2 vs 15.7 per 100 person-years [57].

One study assessed the immunogenicity and safety of the nonavalent vaccine vs placebo [58]. This trial included 313 MSM aged 16–26 years in addition to 1,106 MSW and 1,101 women who had neither received previous HPV vaccination nor had history of HPV-related lesions. The per-protocol analysis showed that, in MSM, the seroconversion rate for different HPV types was 99.5–100%; the GMTs in MSM were lower compared with MSW and women (ratio of 0.6–0.8 and 0.7–0.9, respectively, depending on the genotype analysed) [58]. Data on the efficacy of HPV vaccination for prevention of lesions are not available. No specific data on the safety of HPV vaccines in the MSM population were described in most of the studies evaluated.

#### Summary of previous guidelines or consensus recommendations

The Advisory Committee on Immunisation Practices (ACIP) recommends routine HPV vaccination for MSM aged 9–26 years, including transgender persons [20].

#### Recommendations in men who have sex with men

HPV vaccination is recommended in MSM aged 9–26 years (quality of evidence: moderate; recommendation: strong). MSM aged 26 years and older may also benefit from HPV vaccination (quality of evidence: very low; recommendation: weak). The use of any of the available vaccines is indicated, although the quadrivalent or nonavalent vaccines are preferred given the high prevalence of anogenital warts in MSM.

### Inflammatory bowel disease

Treatment of inflammatory bowel disease IBD (Crohn’s disease and ulcerative colitis) often includes immunosuppressants, which increase the risk of HPV persistence. Compared with patients not receiving immunosuppressive treatment, patients with IBD receiving azathioprine show a higher incidence of cutaneous warts and skin and genital herpes flares [59]. Although some studies have reported the development or worsening of genital condyloma in patients with IBD treated with anti-tumour necrosis factor (TNF) antibodies such as infliximab and etanercept [60,61], other studies suggest no relationship between the use of TNF antibodies and persistent HPV infection [62].

Controversy remains regarding to the increased risk of HPV infection and UCC in patients with IBD vs the general population. A study of 40 patients with IBD showed a higher incidence of abnormal cervical cytology in women with IBD compared with similar healthy controls (42% vs 7%; p < 0.001), and a higher incidence of lesions [63]. In this study, the use of immunosuppressants for at least 6 months was also associated to an increased risk of abnormal cytology (Odds ratio (OR): 1.5; 95% confidence interval (CI): 1.2–4.1; p value = 0.021) [63]. A study of 116 women with IBD showed an increased rate of abnormal cervical cytology [64], while a population-based study of patients with Crohn’s disease demonstrated an increased risk of UCC and dysplasia [65]. Another study showed an increased risk of both low-grade squamous intraepithelial lesions (LSIL), HSIL and UCC in patients with IBD vs a control group (incidence rate ratio for UCC in Crohn’s disease: 1.53; 95% CI: 1.04–2.27) [66]. Case–control studies have not shown an increased risk of histological or cytological cervical abnormalities in women with IBD [67,68].

#### Rationale for recommendations

One paper of HPV vaccination in IBD population was selected (Supplementary material Table S3). One study investigating the profile of the qHPV vaccine in patients with IBD showed an adequate immunogenicity and safety profile [69]. Similar antibody titres were induced in patients with IBD and healthy controls, although 49% of the IBD patients were receiving immunosuppressants and 51% were receiving anti-TNF antibodies. Some major adverse events were detected (exacerbations of IBD (n = 2), pneumonia (n = 1), ovarian torsion (n = 1), acute sinus pain (n = 1)), but none of them appeared to be related to HPV vaccination.

#### Summary of previous guidelines or consensus recommendations

All guidelines regarding prevention of opportunistic infections in patients with IBD recommend regular clinical screening and HPV vaccination, particularly in patients receiving immunosuppressants [70-73].

#### Recommendations in inflammatory bowel disease patients

HPV vaccination is recommended for women with IBD aged 9–26 years (quality of evidence: low; recommendation: weak). Although vaccination can be administered at any time, it is preferable to vaccinate at the time of diagnosis and before the use of immunosuppressive or biological therapies, to ensure higher immunogenicity. There are no data supporting HPV vaccination in men with IBD.

### Women with HPV infection and precancerous cervical lesions

In Spain, a large-scale study estimate that the prevalence of HPV infection is 13.4% and 7.9% in women aged 26–45 and 46–65 years, respectively, compared with 28.8% in women aged 18–25 years [74]. Rates of precancerous cervical lesions are reported between 0.4 and 0.7%; the estimated number of women aged 18 years or older with HSIL (CIN) in Spain is in the range of 54,087–92,423 [75].

#### Rationale for recommendations

A substantial proportion of adult women become infected with HPV, particularly genotypes 16 and 18. The probability of persistence increases with age, which consequently increases the risk of precancerous lesions or UCC developing. Women with precancerous cervical lesions have a 5–25% risk of recurrence and an increased risk of developing UCC compared with the general population [76]. This increased risk is mainly due to persistent HPV. Moreover, while in general the HPV infection clears after treatment, natural immunity does not provide complete protection against reactivation or reinfection by the same HPV genotype [75].

Three papers of HPV vaccination in women with HPV infection and precancerous cervical lesions were selected (Supplementary material Table S4). Precancerous lesions are generally diagnosed in women aged 25 years or older; data about HPV vaccination in this group of women are limited. However, a few studies focused on adult women and showed that both qHPV vaccine and the bivalent HPV vaccine are immunogenic, effective and safe in this age group [77,78]. Immunisation with the qHPV vaccine resulted in 64.9% (95% CI: 20.1–86.3) efficacy in preventing new cervical lesions by any HPV genotype in treated women [79]. Another study of 737 women undergoing cervical conisation showed that the qHPV vaccine was associated with a 65% reduction in HPV recurrence at 2 years, regardless of genotype (2.5% vs 7.2% recurrences in vaccinated and unvaccinated women, respectively) [80]. The post-treatment risk reduction of new lesions following bivalent vaccine immunisation in women with surgically treated cervical lesions was 88.2% (95% CI: 14.8–99.7), regardless of genotype [81]. These studies demonstrate that HPV vaccination in women with cervical lesions who undergo treatment results in a significant reduction in the risk of new lesions. No specific safety data on the HPV vaccines in these populations was identified in any of the studies evaluated.

#### Summary of previous guidelines or consensus recommendations

In Spain, a consensus guideline from several scientific societies recommends HPV vaccination in sexually active women. Because of the increased risk of HPV infection and increased persistence of infection with age in women with prior HPV infection and viral clearance, the intention of this recommendation is to protect against reinfection and to reduce the risk of developing new lesions and UCC in women receiving treatment for cervical lesions [75].

#### Recommendations in women with HPV infection and precancerous cervical lesions

HPV vaccination is recommended in women undergoing treatment for precancerous cervical lesions (quality of evidence: moderate; recommendation: strong). Patients with precancerous cervical lesions who have not yet been treated, may benefit from HPV vaccination (quality of evidence: low; recommendation: strong). Ideally, the vaccine should be administered early, either at diagnosis or before cervical conisation.

### Congenital bone marrow failure syndrome, primary immunodeficiency or survivors of childhood neoplasia

Congenital bone marrow failure syndrome (CBMFS) is a group of heterogeneous diseases associated with inadequate blood cell production, constitutional malformations and predisposition to cancer. Patients with Fanconi’s anaemia and dyskeratosis congenita, both congenital bone marrow failure syndromes, are at risk of gynaecological and head and neck squamous cell carcinomas [82]. The increased risk of cancer in these patients means that cancer prevention strategies are essential.

Certain primary immunodeficiency conditions, such as epidermodysplasia verruciformis, hyper-IgE syndrome, idiopathic CD4 lymphopaenia, GATA2 deficiency and Warts-hypogammaglobulinaemia, infections and myelokathesis (WHIM) or Netherton’s syndrome, have the presence of cutaneous warts as one of the main clinical manifestations of the disease [83]. Patients with these conditions are more susceptible to persistent HPV infection because of an impaired immune response to the virus, an increased risk of extensive skin involvement and a higher risk of precancerous cervical lesions progressing to malignancy.

Survivors of childhood, adolescent or young adulthood cancer e.g. Hematopoietic stem cell transplantation (HSCT) recipients, post-transplant survivors with chronic graft-vs-host disease receiving immunosuppressive therapy, individuals with a history of pelvic radiation and those with Hodgkin’s lymphoma, are also at risk of adverse outcomes associated with HPV infection [84]. In one US study, male and female long-term cancer survivors had 150% and 40% more HPV-related malignancies, respectively, than the general population [85].

#### Rationale for recommendations

Two papers of HPV vaccination in CBMFS, primary immunodeficiency or survivors of childhood neoplasia were selected (Supplementary material Table S5). Patients with Fanconi's anaemia and dyskeratosys congenita have a similar immune response to HPV vaccination to that of the general population [86].

While live-attenuated vaccines are not feasible in patients with primary immunodeficiency, inactivated or subunit vaccines are considered safe [87] and could play an important role in the prevention of both malignancy and warts. Immunisation of a WHIM patient with the qHPV vaccine induced neutralising antibodies and a cellular immune response, indicating that patients with WHIM syndrome may benefit from HPV vaccination [88]. No safety data were reported in any of the studies analysed. Although most survivors of childhood neoplasia recover their immunity within 6 months of the end of chemotherapy or immunosuppressive therapy, the immunogenicity to HPV vaccines in this population is not well known so additional studies are needed.

#### Summary of previous guidelines or consensus recommendations

ACIP recommends routine HPV vaccination to primary immunodeficiency patients aged 9–26 years [20]. The Children’s Oncology Group recommends HPV vaccination for all female survivors of childhood cancer [89,90].

#### Recommendations in patients with congenital bone marrow failure syndrome, primary immunodeficiency or survivors of childhood neoplasia

HPV vaccination of patients with CBMFS is recommended (quality of evidence: low; recommendation: strong).

HPV vaccination may benefit patients with primary immunodeficiency (quality of evidence: very low; recommendation: weak). The use of any of the available vaccines is indicated, although the quadrivalent or nonavalent vaccines are preferred given the high prevalence of anogenital warts in these patients.

Survivors of childhood neoplasia can benefit from HPV vaccination (quality of evidence: very low; recommendation: weak).

### Solid organ or haematopoietic stem cell transplantation

Patients who have undergone solid organ transplantation need life-long immunosuppressive therapy, which increases their risk of complications associated with persistent HPV infection. Studies have shown that immunosuppressed transplant recipients have an increased presence of HPV in the oral cavity, higher prevalence of genital warts [91], high oncogenic HPV in squamous cell skin cancer [92], an increased risk of anal cancer [93-95] and UCC [96-98].

Patients who have undergone HSCT have an increased risk of developing solid organ cancer [99]. In some studies, this risk was double that of the general population [100,101]. The increase in survival after HSCT has been associated with an increased prevalence of HSIL in these women [102,103].

#### Rationale for recommendations

Three papers of HPV vaccination in solid organ or haematopoietic stem cell transplantation were selected [104-106] (Supplementary material Table S6). Epidemiological data show a high risk of gynaecological and other cancers in transplant recipients [102]. Limited data are available on the immunogenicity and efficacy of HPV vaccination in individuals who have undergone solid organ transplantation, and no information exists for HSCT recipients.

#### Summary of previous guidelines or consensus recommendations

ACIP recommends routine HPV vaccination for those aged 9–26 years for transplant patients [20].

The 2013 Infectious Diseases Society of America (IDSA) clinical practice guideline for vaccination of the immunocompromised host recommends that physicians consider the administration of three doses of HPV vaccine 6–12 months after HSCT for female patients aged 11–26 years and qHPV vaccine for males aged 11–26 years. The HPV vaccine series should also be administered to solid organ transplant candidates aged 11–26 years [27].

#### Recommendations in solid organ or haematopoietic stem cell transplant recipients

HPV vaccination is recommended in female transplant recipients aged 9–26 years (quality of evidence: low; recommendation: strong). Female patients aged 26 years and older may also benefit from HPV vaccination, particularly those with chronic graft vs host disease following HSCT (quality of evidence: very low; recommendation: weak). If possible, vaccination should be provided before solid organ transplantation and preferably before the use of immunosuppressive or biological therapies to ensure high immunogenicity. Vaccination is not recommended within 6 months post-transplantation. Patients undergoing HSCT should be vaccinated 6–12 months after transplantation, even if vaccinated beforehand.

### Immunosuppressive or biological treatment

Immunosuppression is an important risk factor for cervical HPV infection [107]. HPV has a predilection for epithelial cells and causes an abnormal proliferation of keratinocytes, which may manifest as benign warts or malignant neoplasms. The host immune response to HPV infection is initiated by cytokines and chemokines that are secreted by the virally stimulated keratinocyte. If the immune response is sufficient HPV lesions may regress, but this resolution of HPV lesions is not generally seen in the immunosuppressed patients resulting in severe, persistent and extensive manifestations of HPV disease [108]. Among patients undergoing therapeutic immunosuppression, evidence is restricted to some disease types e.g. systemic lupus erythematosus (SLE) or juvenile idiopathic arthritis (JIA) and rarely stratified by therapeutic modality or dosage.

Some studies have shown an increase in the prevalence of HPV infection, dysplasia and premalignant lesions in patients with SLE and those with kidney failure undergoing haemodialysis [107,109]. A systematic review of studies in patients with SLE concluded that they are at higher risk of cervical dysplasia and precancerous lesions. However, most studies show a similar prevalence of UCC in patients with SLE compared with the general population [110]. A delay in HPV clearance and a greater persistence of infection and HPV-associated lesions in SLE patients could be explained by alterations in the innate and adaptive immune response in this population [111]. However, the role of immunosuppressants as a predisposing factor to HPV-associated cervical lesions is unconfirmed [102,112].

TNF blockers are widely used to treat chronic inflammatory diseases. TNF plays an essential part in host defence and the immune response. Although uncommon, cases of extensive cutaneous HPV lesions have been reported following TNF blocking therapy [61,113,114].

#### Rationale for recommendations

The high rates of HPV infection and associated lesions in immunocompromised individuals justify the need to consider vaccination in this population [107]. Although published data are limited to some groups of patients, evidence suggests that women with SLE are at an increased risk of developing HPV-related lesions. As SLE is an immune disorder that predominantly affects women, most subjects in studies evaluating the risk of HPV infection and lesions in SLE patients are women [110].

Five papers of HPV vaccination in patients with immunosuppressive or biological treatment were selected. (Supplementary material Table S7) [115-119]. In the largest study, administration of the qHPV vaccine resulted in similar seroconversion rates to the general population 12 months post-vaccination [115]. Lower seroconversion rates were seen in patients receiving treatment with prednisolone or mycophenolate mofetil. There were no safety issues or an increased risk of SLE flares seen with vaccination [115].

Two studies evaluating the immunogenicity and safety of HPV vaccines in JIA patients have been published [118,119]. One was a prospective cohort study including 68 females aged 12–18 years with JIA who were vaccinated with bivalent HPV vaccine. The vaccine showed a strong HPV 16/18-specific antibody response that was comparable with the response seen in healthy female adolescents; HPV-specific geometric mean concentrations (GMCs) were consistently lower in JIA patients. No effect of methotrexate or anti-TNF treatment on the vaccine response was observed and no safety concerns were found [118]. Another prospective cohort study including 21 female patients with JIA aged 12–25 years and 21 healthy controls immunised with bivalent HPV vaccine showed significantly lower HPV 16 neutralising antibody titres in JIA patients than controls, but no significant difference in HPV-18 antibody titres. Immunoresponse to the vaccine did not seem to be affected by the type of anti-rheumatic therapy the patients were receiving. The vaccine was safe and well tolerated [119].

At present, there are no published data from randomised clinical trials on the efficacy, immunogenicity or safety of HPV vaccination in patients receiving immunosuppressive and/or biological treatment.

#### Summary of previous guidelines or consensus recommendations

The evidence-based recommendations on vaccination in patients with autoimmune rheumatic diseases from the European League against Rheumatism specify that HPV vaccination should be considered in women with SLE aged less than 26 years [120].

ACIP recommends routine HPV vaccination for immunosuppressed patients aged 9–26 years [20].

The 2013 IDSA clinical practice guideline for vaccination of the immunocompromised host recommends HPV vaccination for females and males 11–26 years old with chronic inflammatory diseases on immunosuppressive medications [27].

#### Recommendations in patients receiving immunosuppressive or biological treatment

HPV vaccination is recommended in women receiving immunosuppressive and/or biological treatment aged 9–26 years, in particular, women with SLE or JIA (quality of evidence: low; recommendation: weak). While the vaccine can be given at any time, administration before the use of immunosuppressive or biological therapies is preferable. There are no data supporting HPV vaccination in males receiving immunosuppressive or biological therapy.

### Recurrent respiratory papillomatosis

Recurrent respiratory papillomatosis (RRP) is characterised by the presence of multiple papilloma in the airway that cause obstructive symptoms such as shortness of breath, hoarseness or stridor [121]. The disease may occur in children (aged ≤ 17 years) or adults [9,122]. The incidence of both juvenile and adult forms of RRP is estimated at 3–7 per 100,000 individuals [123,124]. HPV infection is the cause of RRP, with genotypes 6 and 11 present in 90–95% of cases [9].

#### Rationale for recommendations

Nine papers of HPV vaccination in patients with recurrent respiratory papillomatosis were selected (Supplementary material Table S8). No randomised clinical trials on the efficacy of HPV vaccine have been conducted in individuals with RRP. Several studies have investigated the effectiveness of the quadrivalent vaccine in both juvenile RRP [122,125-131] and adult RRP [122,126,130,132] (Supplementary material Table S8). Vaccination-induced partial or complete remission in a substantial proportion of the RRP patients, and no remarkable adverse effects were reported.

#### Summary of previous guidelines or consensus recommendations

There are no guideline or consensus documents that specifically recommend HPV vaccination in patients with RRP.

#### Recommendations in patients with RRP

HPV vaccination is recommended in patients with RRP aged 9–26 years (quality of evidence: low; recommendation: strong). Given the relationship of RRP to HPV genotypes 6 and 11, the use of the quadrivalent or nonavalent vaccine is recommended. Despite scarce data, the potential benefit should outweigh the risk of vaccination; therefore compassionate use may be considered in juvenile patients who are under the age indicated in the summary of product characteristics for the vaccine.

### Summary of recommendations

Data concerning non-routine HPV vaccination in populations with a high risk of HPV infection and associated lesions are scarce, and recommendations differ between countries. The evidence-based recommendations for HPV vaccination in high-risk populations conducted by an independent expert panel are summarised in Table.

**Table:** Summary of recommendations regarding human papillomavirus vaccination in high-risk populations

High-risk population	Summary and recommendations	Quality of evidence	Recommendation strength
HIV infection	HPV vaccination is recommended in HIV patients through age 26, regardless of gender	Moderate	Strong
	HIV-positive patients aged ≥ 26 years may also benefit from HPV vaccination	Low	Weak
MSM	HPV vaccination in MSM through age 26 is recommended	Moderate	Strong
	MSM aged ≥ 26 years may benefit from HPV vaccination	Very Low	Weak
IBD	HPV vaccination is recommended in women with IBD through age 26	Low	Weak
Women with premalignant cervical lesions	HPV vaccination is recommended in women treated for precancerous cervical lesions	Moderate	Strong
	Women with untreatable intraepithelial lesions may benefit from HPV vaccination	Low	Strong
CBMFS	HPV vaccination is recommended in patients with CBMFS	Low	Strong
Primary immunodeficiency	Patients with primary immunodeficiency may benefit from HPV vaccination	Very low	Weak
Survivors of childhood neoplasia	Survivors of childhood cancer may benefit from HPV vaccination	Very low	Weak
Solid organ transplantation or HSCT	HPV vaccination is recommended in women through age 26 who have received a solid organ transplant or HSCT	Low	Strong
	Female patients aged ≥ 26 years may benefit from HPV vaccination, particularly those with chronic graft-vs-host disease after HSCT	Very low	Weak
Immunosuppressive or biological treatment	HPV vaccination is recommended in women receiving immunosuppressive and/or biological treatment through age 26 (in particular women with SLE or JIA)	Low	Weak
RRP	HPV vaccination is recommended in patients diagnosed with RRP through age 26	Low	Strong

CBMFS: congenital bone marrow failure syndrome; HIV: human immunodeficiency virus; HPV: human papillomavirus; HSCT: haematological stem cell transplantation; IBD: inflammatory bowel disease; JIA: juvenile idiopathic arthritis; MSM: men who have sex with men; RRP: recurrent respiratory papillomatosis; SLE: systemic lupus erythematosus.

## Conclusions

Systematic HPV vaccination is considered the most effective intervention for controlling the infection and preventing HPV-related disease burden. Along with other interventions, such as information, screening and treatment, HPV vaccination is crucial in the prevention of uterine cervical cancer.

In all high-risk populations, based on the summary and recommendations, the quality of evidence was found to be very low to moderate. A strong recommendation about HPV vaccination was made in HIV infected patients aged 9–26 years; men who have sex with men aged 9–26 years; women with precancerous cervical lesions; patients with congenital bone marrow failure syndrome; women who have received a solid organ transplant or hematopoietic stem cell transplantation aged 9–26 years; and patients diagnosed with recurrent respiratory papillomatosis. In the rest of high-risk categories with only a weak recommendation (HIV-positive patients aged ≥ 26 years, MSM aged ≥ 26, IBD, patients with primary immunodeficiency, survivors of childhood neoplasia, women who have received a solid organ transplant or hematopoietic stem cell transplantation aged ≥26 years and patients in immunosuppressive or biological treatment), the quality of evidence was low or very low. Further studies are needed.

## Note

Author Xavier Castellsagué passed away 12 June 2016.

## Supplementary Data

672000 Supplementary Material S1
