# Supplementary material for: Multidisciplinary, evidence-based consensus guidelines for human papillomavirus (HPV) vaccination in high-risk populations, Spain, 2016
Source: Euro Surveill. 2019 Feb 14;24(7):1700857. doi: 10.2807/1560-7917.ES.2019.24.7.1700857 (PMC6381660; doi:10.2807/1560-7917.ES.2019.24.7.1700857)
Supplement: Supplementary Material S1 [file 1700857_CAMPINS_SupplementaryMaterial.pdf]

Supplementary Material S1

This supplementary material is hosted by Eurosurveillance as supporting information alongside the article [Multidisciplinary,evidence-based consensus guidelines for human papilloma virus (HPV) vaccination in high-risk populations, Spain, 2016] on behalf of the authors who remain responsible for the accuracy and appropriateness of the content. The same standards for ethics, copyright, attributions and permissions as for the article apply. Eurosurveillance is not responsible for the maintenance of any links or email addresses provided therein.

General considerations to HPV vaccination in high-risk groups

• No preferences between any of the licensed vaccines available have been established, except when indicated.

• No current evidence supports vaccination schedules with fewer than 3 doses in high-risk populations.

• Long-term studies evaluating the duration of vaccine protection are lacking.

• No minimum threshold of antibody Geometric Mean Titres (GMT) has been internationally established for protection against HPV, even though some studies have used their own standards.

• Data was consistently limited to people under 26 years, especially in women, which may be related to two factors: vaccine licensing criteria (first authorised in women aged less than 26 years), and cervical cancer as the main reason for vaccination; in people living with HIV there was no limitation regarding age for reviewing published data. However, considering other high-risk populations, the high impact of HPV and developing HPV-related diseases, improvements in efficacy may represent a significant individual benefit, despite characteristics, such as sex, age and type of related lesions. Moreover, even though there is a paucity of robust data on safety in most groups, data in women from the general population aged ≥ 30 years confirm safety and tolerability of available HPV vaccines [1-3]. The European Medicines Agency (EMA) approved increasing the indicated age for HPV vaccination in women, with no age limits for the prevention of certain cancers. Similarly, the US Food and Drug Administration (FDA) is currently considering increasing the indicated age for HPV vaccination for women from 27 to 45 years old.

• The data on immunogenicity, efficacy and safety of HPV vaccines in patients aged 26 years or above in subgroups of high-risk populations are scarce. Specific recommendations for these individuals could not be established, despite the likelihood of benefit from HPV vaccination

References:

1. GlaxoSmithKline. Cervarix suspension for injection: summary of product characteristics. GlaxoSmithKline; 2012. Available from: http://www.ema.europa.eu/docs/en_GB/document_library/EPAR_-_Product_Information/human/000721/WC500024632.pdf</eref>
2. Merck Sharp & Dohme BV. Gardasil suspension for injection: summary of product characteristics. Merck Sharp & Dohme BV; 2011. Available from: http://www.ema.europa.eu/docs/en_GB/document_library/EPAR_-_Product_Information/human/000703/WC500021142.pdf</eref>
3. Merck Sharp & Dohme BV. Gardasil 9 suspension for injection: summary of product characteristics. Merck Sharp & Dohme BV; 2015. Available from: http://www.ema.europa.eu/docs/en_GB/document_library/EPAR_-_Product_Information/human/003852/WC500189111.pdf

Table S1. Published studies investigating human papillomavirus vaccination in populations with HIV, January 2006 to June 2016 (n = 9)

Citation	Study design	Population (n)[a]	Vaccine type	Seroconversion (%)	Safety	Comments
Levin et al., 2010 [1]	Randomised, multicentre, placebo controlled	Children aged 7–12 years (126)	Quadrivalent	97–100	Any AE: 36% vaccine and 50% placebo;<br>8 subjects G3 and 2 subjects G4 AE, all not vaccine-related.<br>AE not differ depending on CD4.	GMT was lower than age-matched historical cohorts of HIV-negative patients (30–50% less for HPV 6 and 18).
Wilkin et al., 2010 [2]	Prospective, multicentre, single-arm, cohort	Males aged 22–61 years (112)	Quadrivalent	95–100	No ≥ G3 AE.<br>G2 local reaction: 8%.<br>Other G2 vaccine-related: 5%.<br>1 death unrelated to vaccine.	GMT 40% lower than in historic cohorts of young men and women without HIV but similar to HPV MSM titres [36]; ART at the time of vaccination associated with higher antibody titres.
Kahn et al., 2013 [3]	Prospective, multicentre, single-arm cohort study	Women aged 16–23 years (99; 30 not receiving ART)	Quadrivalent	92–100% (-ART)<br>100% (+ ART)	48.5% ≥ 1 local/systemic AE.<br>Local reaction 26.3% (all G1 except 1 G2).<br>Systemic AE 24.2% (headache 15.2%, fever 12.1%).<br>1 severe AE (G3 fatigue).	Compared with historic cohort of HIV-negative patients, GMT was similar in patients receiving ART but lower in patients not receiving ART.
Denny et al., 2013 [4]	HIV + :<br>Randomised, placebo-controlled<br>HIV-: Prospective single-centre, single-arm cohort	Women aged 18–25 years,<br>HIV + (120) and HIV- (30)	Bivalent	100% in vaccinated patients (all HIV-cohort and the treatment arm in HIV + study)	G3 local pain in 1.9% doses in HIV + and 1.2% doses in HIV-.<br>G3 systemic AE in no more than 0.6% doses in all groups.<br>Any AE 30-day FU 86.9% in HIV + and 86.7% in HIV- .<br>No D/C due to AE. No deaths.	Baseline seropositivity for HPV 16 and 18: 85% and 64% in HIV + ; 64% and 50% in HIV-.<br>Lower GMT titres in HIV + (ca 50–70%).<br>No impact on CD4 titres or baseline viral load in post-vaccine response.
Giacomet et al., 2014 [5]	Non-randomised, single-centre	Adolescents and young adults (age 13–27 years), of both sexes,<br>HIV + (46) and HIV- (46)	Quadrivalent	85% HIV +<br>91% HIV-	Local AE: 64.5% HIV + , 41.9% HIV-.<br>Systemic AE: 23.4% HIV + , 3.6% HIV-.	No statistically significant differences in antibody titres.

Kojic et al., 2014 [6]	Prospective, multicentre, single-arm cohort	Women aged 13–45 years (319)	Quadrivalent	> 75% for all types	17% G ≥ 3 AE. 16 G ≥ 1 fever (1 G3). 3 G2 local reactions. 2 deaths not vaccine-related.	Lower seroconversion rate in patients with HIV viral load > 10,000 copies/mL and/or CD4 < 200 cells/mm^3. GMT HPV 16/18 ca 50%, lower when compared with a historic cohort of HIV- women. Only study including patients with CD4 < 200 cells/mm^3.
Toft et al., 2014 [7]	Randomised, single-centre, double-blind, active-controlled	Men and women aged 22–72 years (92)	Bivalent vs quadrivalent	Not reported	Local reactions: bivalent 91.1%, quadrivalent 69.6%, without gender differences. No serious AE.	Bivalent: 30 men/15 women. Quadrivalent: 31 men/15 women. Higher anti-HPV-18 titres with the bivalent vs quadrivalent vaccine both in men and women. No significant differences in anti-HPV-16 titres among vaccine groups. Higher GMT (HPV 16 and 18) in women vs men with bivalent but not with quadrivalent vaccine.
Money et al., 2016 [8]	Prospective, multicentre, single-arm, open-label cohort	Women aged > 15 years, age range 15–66 years (372)	Quadrivalent	94–99%	36% ≥ 1 AE. Local reactions 31%. Systemic AE 20%. 1 serious AE possibly related to vaccine (encephalopathy). 2 deaths, not vaccine-related.	Significantly higher (1.74–3.05 fold) peak antibody response in patients with undetectable HIV RNA.
Fontes et al., 2016 [9]	Prospective, single-centre, single-arm cohort	Men aged 18–45 years (19)	Quadrivalent	17 out of 19 patients	No serious AE reported.	Similar increase of circulating antibodies in people with serum CD4 levels < 500 cells/mm^3 (8/9) and ≥ 500 cells/mm^3 (9/10).

AE: adverse events; ART: antiretroviral treatment; D/C: discontinuations; FU: follow-up; G: grade (1–4); GMT: geometric mean concentrations of antibodies; HIV: human immunodeficiency virus; HIV-: HIV negative; HIV + : HIV positive; HPV: human papillomavirus.

[a]Population was all HIV positive unless otherwise stated.

References:

1. Levin MJ, Moscicki AB, Song LY, Fenton T, Meyer WA 3rd, Read JS, et al.; IMPAACT P1047 Protocol Team. Safety and immunogenicity of a quadrivalent human papillomavirus (types 6, 11, 16, and 18) vaccine in HIV-infected children 7 to 12 years old .J Acquir Immune Defic Syndr. 2010;55(2):197-204. http://dx.doi.org/10.1097/QAI.0b013e3181de8d26 PMID:20574412</jrn>

2. Wilkin T, Lee JY, Lensing SY, Stier EA, Goldstone SE, Berry JM, et al. Safety and immunogenicity of the quadrivalent human papillomavirus vaccine in HIV-1-infected men. J Infect Dis. 2010;202(8):1246-53. http://dx.doi.org/10.1086/656320 PMID:20812850

3. Kahn JA, Xu J, Kapogiannis BG, Rudy B, Gonin R, Liu N, et al. Immunogenicity and safety of the human papillomavirus 6, 11, 16, 18 vaccine in HIV-infected young women. Clin Infect Dis. 2013;57(5):735-44. http://dx.doi.org/10.1093/cid/cit319 PMID:23667266

4. Denny L, Hendricks B, Gordon C, Thomas F, Hezareh M, Dobbelaere K, et al. Safety and immunogenicity of the HPV-16/18 AS04-adjuvanted vaccine in HIV-positive women in South Africa: a partially-blind randomised placebo-controlled study. Vaccine. 2013;31(48):5745-53. http://dx.doi.org/10.1016/j.vaccine.2013.09.032 PMID:24091311

5. Giacomet V, Penagini F, Trabattoni D, Viganò A, Rainone V, Bernazzani G, et al. Safety and immunogenicity of a quadrivalent human papillomavirus vaccine in HIV-infected and HIV-negative adolescents and young adults. Vaccine. 2014;32(43):5657-61. http://dx.doi.org/10.1016/j.vaccine.2014.08.011 PMID:25149430

6. Kojic EM, Kang M, Cespedes MS, Umbleja T, Godfrey C, Allen RT, et al. Immunogenicity and safety of the quadrivalent human papillomavirus vaccine in HIV-1-infected women. Clin Infect Dis. 2014;59(1):127-35. http://dx.doi.org/10.1093/cid/ciu238 PMID:24723284

7. Toft L, Storgaard M, Müller M, Sehr P, Bonde J, Tolstrup M, et al. Comparison of the immunogenicity and reactogenicity of Cervarix and Gardasil human papillomavirus vaccines in HIV-infected adults: a randomized, double-blind clinical trial. J Infect Dis. 2014;209(8):1165-73. http://dx.doi.org/10.1093/infdis/jit657 PMID:24273179

8. Money DM, Moses E, Blitz S, Vandriel SM, Lipsky N, Walmsley SL, et al.; HPV in HIV Study Group. HIV viral suppression results in higher antibody responses in HIV-positive women vaccinated with the quadrivalent human papillomavirus vaccine. Vaccine. 2016;34(40):4799-806. http://dx.doi.org/10.1016/j.vaccine.2016.08.016 PMID:27544584

9. Fontes A, Andreoli MA, Villa LL, Assone T, Gaester K, Fonseca LAM, et al. High specific immune response to a bivalent anti-HPV vaccine in HIV-1-infected men in São Paulo, Brazil. Papillomavirus Res. 2016;2:17-20. http://dx.doi.org/10.1016/j.pvr.2016.01.001 PMID:29074177

TABLE S2. Published studies investigating human papillomavirus vaccination in MSM populations, January 2006 to June 2016 (n = 6)

Citation	Study design	Population (n)	Vaccine type	Seroconversion (%)	Safety	Comments
Palefsky et al., 2011 [1]	Randomised, multicentre, placebo controlled	MSM aged 16-26 (602)	Quadrivalent	Not reported	Any AE at injection site vaccine-related: 58% vaccine and 59% placebo;<br>Any systemic AE vaccine-related: 18% vaccine and 19% placebo<br>2 Serious AE in vaccination group, none related to vaccine	Vaccine Efficacy against HPV-6, 11, 16, or 18–related anal intraepithelial Neoplasia:<br>- ITT analysis: 50.3% (95% CI, 25.7 to 67.2);<br>- PP analysis: 77.5% (95% CI, 39.6 to 93.3)<br>Vaccine efficacy against HPV-6, 11, 16, or 18 persistent anal Infection:<br>- ITT analysis: 59.4% (95% CI, 43.0 to 71.4)<br>- PP analysis: 94.9% (95% CI, 80.4 to 99.4)
Giuliano et al., 2011 [2]	Randomised, multicentre, placebo controlled	Men aged 16-26 (4065):<br>- HM, aged 16-23 (3463)<br>- MSM, aged 16-26 (602)	Quadrivalent	No stratified analysis was made according to sexual orientation<br><br>At least 97.4% of vaccinated people seroconverted	No stratified analysis was made according to sexual orientation<br><br>Any AE at injection site vaccine-related: 60% vaccine and 54% placebo;<br>Any systemic AE vaccine-related: 14% vaccine and 15% placebo<br>Serious AE: 8 cases in vaccine group; 11 cases in placebo group, none related to vaccine	Vaccine efficacy against external genital lesion:<br>- ITT analysis: 70.2 (23.0 to 90.2)<br>- PP analysis: 79.0 (−87.9 to 99.6)<br><br>Vaccine efficacy against Persistent Infection with HPV Type 6, 11, 16, or 18 and against Detection of HPV DNA:<br>- ITT analysis: 43.6 (19.5 to 60.8)<br>- PP analysis: 94.4 (64.4, 99.9)
Hillman et al., 2012 [3]	Randomised, multicentre, placebo controlled	Men aged 16-26 (4065):<br>- HM, aged 16-23 (3463)<br>- MSM, aged 16-26 (602)	Quadrivalent	HM vs MSM (HPV genotype):<br><br>Month 7:<br>99 vs 97 (6)<br>99 vs 97 (11)<br>99 vs 94 (16)<br>98 vs 89 (18)<br><br>Month 36:<br>90 vs 80 (6)<br>94 vs 89 (11)<br>98 vs 94 (16)<br>57 vs 54 (18)	Not reported	Heterosexual subjects had higher GMT levels for all vaccine HPV types at their peak (month 7) and at the end of the study (month 36) compared to MSM

Swedish et al., 2012 [4]	Non-concurrent, single centre, cohort study	202 MSM, aged 20-79:<br>- 88 vaccinated<br>- 114 unvaccinated	Quadrivalent	Not reported	Not reported	Incidence rate of recurrent HGAIN: 10.2 per 100 person-years (95%CI: 5.3–17.8/100 person-years) in vaccinated; 15.7 per 100 person-years (95% CI: 10.9–21.9/100 person-years) in unvaccinated people.<br><br>Kaplan–Meier survival analysis demonstrated improved recurrence-free survival of vaccinated persons compared with unvaccinated persons in each year through the 3 years-period.
Swedish et al., 2014 [5]	Post-hoc analysis of non-concurrent, single centre, cohort study	313 MSM, aged >=26 years-old<br>- 116 vaccinated<br>- 197 unvaccinated	Quadrivalent	Not reported	Not reported	Incidence rate of anal condyloma: 3.7 per 100 person-years (95%CI: 1.8–6.8/ 100 person-years) in vaccinated; 7.3 per 100 person-years (95% CI 5.2–10.1/100 person-years) in unvaccinated people.<br><br>Kaplan-Meier survival analysis demonstrated improved condyloma-free survival of vaccinated persons compared with unvaccinated.
Castellsagué et al., 2015 [6]	Prospective, multicentre, open-label cohorts	Men aged 16-26 (1429):<br>- HM (1106)<br>- MSM (313)<br>Women aged 16-26 (1101)	Nonavalent	> 99% for all types	No stratified analysis was made according to sexual orientation in men<br><br>Any AE: 76% men and 89% women<br>Any AE at injection site: 68% men and 84% women<br>Any systemic AE vaccine-related: 16% men and 23% women<br>Serious AE: 23 cases in men cohort; 26 cases in women cohort, none related to vaccine	For all vaccine HPV types, the month 7 GMTs in MSM was lower than those in HM and women.<br><br>The point estimates of the GMT ratios between MSM and the other two groups ranged as follows: 0.7-0.9 in MSM/women; 0.6-0.8 in MSM/HM

AE: adverse events; GMT: geometric mean concentrations of antibodies; HM: Heterosexual males, ITT: Intention-to treat; MSM: Males who have sex with males; PP: Per-protocol; HPV: human papillomavirus

References:

1. Palefsky JM, Giuliano AR, Goldstone S, Moreira ED Jr, Aranda C, Jessen H, et al. HPV vaccine against anal HPV infection and anal intraepithelial neoplasia. N Engl J Med. 2011;365(17):1576-85. http://dx.doi.org/10.1056/NEJMoa1010971 PMID:22029979
2. Giuliano AR, Palefsky JM, Goldstone S, Moreira ED Jr, Penny ME, Aranda C, et al. Efficacy of quadrivalent HPV vaccine against HPV Infection and disease in males. N Engl J Med. 2011;364(5):401-11. http://dx.doi.org/10.1056/NEJMoa0909537 PMID:21288094
3. Hillman RJ, Giuliano AR, Palefsky JM, Goldstone S, Moreira ED Jr, Vardas E, et al. Immunogenicity of the quadrivalent human papillomavirus (type 6/11/16/18) vaccine in males 16 to 26 years old. Clin Vaccine Immunol. 2012;19(2):261-7. http://dx.doi.org/10.1128/CVI.05208-11 PMID:22155768

4. Swedish KA, Factor SH, Goldstone SE. Prevention of recurrent high-grade anal neoplasia with quadrivalent human papillomavirus vaccination of men who have sex with men: a nonconcurrent cohort study. Clin Infect Dis. 2012;54(7):891-8. http://dx.doi.org/10.1093/cid/cir1036 PMID:22291111

5. Swedish KA, Goldstone SE. Prevention of anal condyloma with quadrivalent human papillomavirus vaccination of older men who have sex with men. PLoS One. 2014;9(4):e93393. http://dx.doi.org/10.1371/journal.pone.0093393 PMID:24714693

6. Castellsagué X, Giuliano AR, Goldstone S, Guevara A, Mogensen O, Palefsky JM, et al. Immunogenicity and safety of the 9-valent HPV vaccine in men. Vaccine. 2015;33(48):6892-901. http://dx.doi.org/10.1016/j.vaccine.2015.06.088 PMID:26144901

TABLE S3. Published studies investigating human papillomavirus vaccination in patients with IBD, January 2006 to June 2016 (n = 1)

Citation	Study design	Population (n)	Vaccine type	Seroconversion (%)	Safety	Comments
Jacobson et al., 2013 [1]	Prospective, multicentre, single-arm, open-label cohort	Two cohorts:<br>- Prospective cohort, of children, adolescents and young adults (age 9–26 years) (n=37)<br>- Previously immunized cohort, of adolescents and young adults (age 14–26 years) (n=15)	Quadrivalent	- Prospective cohort: 92-100%<br>- Previously immunized cohort: 40-100%	AEs: number of patients affected/number of patients with available data<br>• Soreness at injection site: 17/35 after dose 1; 15/32 after dose 2; 17/33 after dose 3<br>• Itchiness: no cases after dose 1 and 2; 2/33 after dose 3<br>• Nausea: 1/35 after dose 1; no cases after dose 2; 3/33 after dose 3<br>• Headache: 3/35 after doses 1, 3/32 after dose 2; 2/33 after dose 3<br>• Fatigue: 1/35 after dose 1; 3/35 after dose 2; 1/33 after dose 3<br>• 5 serious adverse events: Two patients were hospitalized for IBD exacerbations, 1 for pneumonia and 1 for an ovarian torsion secondary to endometriosis.	Both cohorts had a 100% seroconversion to HPV 6, HPV 11 and HPV 16. HPV18 had lower seroconversion rates in both groups (95% in prospective cohort, age 9-15; 92% in prospective cohort age 16-26; 40% in previously immunized cohort, age 14-26)<br>Postvaccination GMT in the prospective cohort was as high or higher to all 4 HPV types compared with a healthy comparison group within each age group

GMT: geometric mean concentrations of antibodies; HPV: human papillomavirus.

Reference:

1. Jacobson DL, Bousvaros A, Ashworth L, Carey R, Shrier LA, Burchett SK, et al. Immunogenicity and tolerability to human papillomavirus-like particle vaccine in girls and young women with inflammatory bowel disease. Inflamm Bowel Dis. 2013;19(7):1441-9

TABLE S4. Published studies investigating human papillomavirus vaccination in women with HPV infection and precancerous cervical lesions, January 2006 to June 2016 (n = 3)

Citation	Study design	Population (n)	Vaccine type	Precancerous cervical lesions	Vaccine efficacy	Safety	Comments
Joura et al., 2012 [1]	Randomised, double-blind, placebo controlled trial (post-hoc analysis)	Women aged 15–26 years Vaccine (n=587) Placebo (n=763)	Quadrivalent	CIN 1 or worse	Reduction of subsequent HPV related disease after cervical surgery: 48.3% (95%CI: 19.1 to 67.6)	Not reported	Follow-up = 30 months
				CIN 2 or worse	Reduction of subsequent HPV related disease after cervical surgery: 64.9% (95%CI: 20.1 to 86.3)	Not reported	Follow-up = 30 months
Kang et al., 2013 [2]	Prospective, single centre, cohort study	Women aged 20–45 years Vaccine (n=360) Control (n=377)	Quadrivalent	CIN 2-3	Recurrence of CIN 2-3 Vaccine group: 2.5% Control group: 7.2%	Not reported	Median follow-up time: 3.5 years
Garland et al., 2016 [3]	Randomized, double-blind, placebo controlled trial (post-hoc analysis)	Women aged 15–25 years Vaccine (n=190) Placebo (n=264)	Bivalent	CIN 1 or worse	Reduction of subsequent HPV related disease after cervical surgery: 42.6% (95%CI: -21.1 to 74.1)	Not reported	Median follow-up: 47 months
				CIN 2 or worse	Reduction of subsequent HPV related disease after cervical surgery: 88.2% (95%CI: 14.8 to 99.7)	Not reported	Median follow-up: 47 months

CIN: Cervical Intraepithelial Neoplasia, HPV: human papillomavirus.

References:

1. Joura EA, Garland SM, Paavonen J, Ferris DG, Perez G, Ault KA, et al.; FUTURE I and II Study Group. Effect of the human papillomavirus (HPV) quadrivalent vaccine in a subgroup of women with cervical and vulvar disease: retrospective pooled analysis of trial data. BMJ. 2012;344(mar27 3):e1401.

http://dx.doi.org/10.1136/bmj.e1401 PMID:22454089

2. Kang WD, Choi HS, Kim SM. Is vaccination with quadrivalent HPV vaccine after loop electrosurgical excision procedure effective in preventing recurrence in patients with high-grade cervical intraepithelial neoplasia (CIN2-3)? Gynecol Oncol. 2013;130(2):264-8. http://dx.doi.org/10.1016/j.ygyno.2013.04.050 PMID:23623831

3. Garland SM, Paavonen J, Jaisamrarn U, Naud P, Salmerón J, Chow SN, et al.; HPV PATRICIA Study Group. Prior human papillomavirus-16/18 AS04-adjuvanted vaccination prevents recurrent high grade cervical intraepithelial neoplasia after definitive surgical therapy: Post-hoc analysis from a randomized controlled trial. Int J Cancer. 2016;139(12):2812-26. http://dx.doi.org/10.1002/ijc.30391 PMID:27541373

TABLE S5. Published studies investigating human papillomavirus vaccination in patients with Congenital bone marrow failure syndrome, primary immunodeficiency or survivors of childhood neoplasia, January 2006 to June 2016 (n = 2)

Citation	Study design	Population (n)	Vaccine type	Seroconversion % (HPV genotype)	Safety	Comments
Handisurya et al., 2010 [1]	Case report	12-year-old female patient diagnosed of WHIM-syndrome	Quadrivalent	HPV-specific antibody titers were measured by ELISA using VLP of HPV 6, 11, 16 or 18 as the antigen. Response was compared with three healthy volunteers.<br><br>- WHIM-syndrome patient: a titer of 400 for HPV 6, HPV 11, HPV 16, and a titer of 100 for HPV 18<br><br>- Controls: antibody titers from 6400 to 102,400 against each type of HPV.	No adverse effects observed during the vaccination period and the follow-up period of 16 months	Cellular immune response was also determined, concluding that the proliferative response to mitogens was conserved in the WHIM-syndrome patient
Katzenellenbogen et al., 2015 [2]	Cross-sectional study	Men and Women aged 3-42 years at the time of serum collection, affected of Fanconi's ananemia (59). Includes both vaccinated and unvaccinated patients	Not reported	Unvaccinated (n=29) vs vaccinated (n=24):<br><br>38 vs 92 (6)<br>24 vs 92 (11)<br>34 vs 96 (16)<br>7 vs 75 (18)	Not reported	Positive serology against nonvaccine HPV types were also analyzed between unvaccinated and vaccinated patients, with the following percentage of seroconversion according to HPV genotype:<br><br>21 vs 71 (1)<br>7 vs 54 (2)<br>14 vs 38 (4)<br>10 vs 50 (52)<br>14 vs 67 (58)

WHIM: Warts-hypogammaglobulinaemia, infections and myelokathesis; HPV: human papillomavirus; Elisa: Enzyme Linked Immunosorbent Assay; VLP: Virus-Like Particles.

References:

1. Handisurya A, Schellenbacher C, Reininger B, Koszik F, Vyhnanek P, Heitger A, et al. A quadrivalent HPV vaccine induces humoral and cellular immune responses in WHIM immunodeficiency syndrome. Vaccine. 2010;28(30):4837-41. http://dx.doi.org/10.1016/j.vaccine.2010.04.057 PMID:20472031
2. Katzenellenbogen RA, Carter JJ, Stern JE, Butsch Kovacic MS, Mehta PA, Sauter SL, et al. Skin and mucosal human papillomavirus seroprevalence in persons with Fanconi Anemia. Clin Vaccine Immunol. 2015;22(4):413-20. http://dx.doi.org/10.1128/CVI.00665-14 PMID:25651924

Table S6. Published studies investigating human papillomavirus vaccination in recipients of solid organ transplantation, January 2006 to June 2016 (n = 3)

Citation	Study design	Population	Vaccine	Seroconversion	Safety	Comments
Kumar et al. 2013 [1]	Prospective, non-randomised cohort	Young adults between 18 and 35 years (n = 47; 31 women)<br>Type of transplant:<br>• Kidney: 30 patients<br>• Lung: 11 patients<br>• Heart: 3 patients<br>• Liver: 1 patient<br>• Other: 2 patients	Quadrivalent<br>Number of vaccine doses received:<br>1. 47 patients<br>2. 45 patients<br>3. 43 patients	38 patients had a serologic test performed 1 month after the third dose of vaccine:<br>• 18 patients responded to all four genotypes of the vaccine<br>• 26 patients responded to genotype 11<br>• 20 patients responded to genotype 18	AEs: number of patients affected/number of patients with available data<br>• Local tenderness: 10/45 after dose 1; 1/45 after dose 2; no cases after dose 3<br>• Fatigue: 4/45 after dose 1; no cases after dose 2 and and dose 3<br>• Fever: 1/45 after doses 1 and 2; no cases after dose 3<br>• Dizziness: 1/45 after dose 1; no cases after dose 2 and dose 3<br>• Death of chronic allograft rejection: 2/43 after dose 3<br>• Anal intraepithelial neoplasia: 1/41 after dose 2<br>LSIL on cervical smears: 3 female patients after dose 3	Lung transplant recipients had a lower response rate to the vaccine than kidney transplant recipients; median time from transplant to vaccination was 3.24 years in responders vs 0.74 years in non-responders

Gómez-Lobo et al. 2014 [2]	Prospective, non-randomised cohort	Adolescents between the ages of 9 and 17 at recruitment (n = 17)<br>Type of transplant:<br>• Kidney: 14<br>• Liver: 3	Quadrivalent<br>Number of vaccine doses received:<br>1: Three patients<br>2: Five patients<br>3: Nine patients	7 kidney + 1 liver transplant recipients had a serologic test performed after dose 2:<br>• 5 kidney + 1 liver transplant recipients responded to all four genotypes of the vaccine<br>7 kidney + 1 liver transplant recipients had a serologic test performed after dose 3:<br>• All responded to all four genotypes of the vaccine	AEs: number of patients affected/number of patients with available data<br>• Fever: 4/17<br>• Swelling and pain at the injection site: 3/17<br>• Acne: 1/17<br>• Cough: 1/17<br>• Pneumonia: 1/17<br>• Diarrhoea: 1/17<br>• Headache: 1/17<br>• Acute rejection in kidney transplant recipients: 6/14; 3 cases after dose 1; 2 cases after dose 2; 1 case after dose 3	Kidney transplant recipients had GMTs for HPV antibodies similar to historic healthy controls, but liver transplant recipients had substantially lower GMTs for anti-HPV 6 and anti-HPV 16
Nelson et al. 2016 [3]	Prospective, non-randomised cohort	Girls with chronic kidney disease (n = 25), dialysis dependence (n = 9) or a history of kidney transplantation (n = 23)	Quadrivalent<br>All 23 patients received 3 doses of the vaccine	22 transplant recipients had a serologic test performed at a median of 3.6 months after dose 3:<br>• 14 responded to genotype 6<br>• 14 responded to genotype 11<br>• 22 responded to genotype 16<br>• 15 responded to genotype 18	Adverse event: number of patients affected/number of patients with available data<br>• Acute rejection: 2/23; one case between dose 2 and 3 and the other case after dose 3	IgG antibody titres were significantly lower in patients with transplants compared to patients with chronic kidney disease, for all four genotypes

AE: adverse events; GMT: geometric mean titres of antibodies; HPV: human papillomavirus; IgG: Immunoglobulin G; LSIL: low-grade squamous intraepithelial lesion.

References:

1. Kumar D, Unger ER, Panicker G, Medvedev P, Wilson L, Humar A. Immunogenicity of quadrivalent human papillomavirus vaccine in organ transplant recipients. Am J Transplant. 2013;13(9):2411-7. http://dx.doi.org/10.1111/ajt.12329 PMID:23837399

2. Gomez-Lobo V, Whyte T, Kaufman S, Torres C, Moudgil A. Immunogenicity of a prophylactic quadrivalent human papillomavirus L1 virus-like particle vaccine in male and female adolescent transplant recipients. Pediatr Transplant. 2014;18(3):310-5. http://dx.doi.org/10.1111/petr.12226 PMID:24484551

3. Nelson DR, Neu AM, Abraham A, Amara lS, Batisky D, Fadrowski JJ. Immunogenicity of Human Papillomavirus Recombinant Vaccine in Children with CKD. Clin J Am Soc Nephrol. 2016;11(5):776-84. http://dx.doi.org/10.2215/CJN.09690915 PMID:27055465

Table S7. Published studies investigating human papillomavirus vaccination in patients with immunosupressive or biological treatment, January2006 to June 2016 (n = 5)

Citation	Study design	Population (n)	Vaccine	Seroconversion % (HPV genotype)	Safety	Comments
Mok et al. 2013 [1]	Case–control	Women aged 18–35 years (50 patients with SLE and 50 healthy controls)	Quadrivalent	82 vs 98 (6)<br>89 vs 98 (11)<br>95 vs 98 (16)<br>76 vs 80 (18)	Incidence of AEs was comparable between patients with SLE and controls.<br>No increase in lupus activity or flares.	None.
Heijstek et al. 2013 [2]	Case–control	Women aged 12–18 years (6 patients with SLE and 49 healthy controls)	Bivalent	All SLE patients were seropositive for HPV16/18 after the third vaccine dose.	Not reported	HPV 16/18-specific antibody concentrations were lower in SLE patients vs controls at month 7 post-vaccination but similar at month 12.
Soybilgic et al. 2013 [3]	Prospective, single-arm cohort	Women aged 12–26 years with SLE (27)	Quadrivalent	94.4 (6)<br>100 (11)<br>100 (16)<br>94.4 (18)	9 patients had mild-moderate flare during the study period, with symptoms similar to those experienced in flares before vaccine administration.	Two patients became pregnant after the second dose of HPV vaccine. Both pregnancies resulted in healthy newborns.
Heijstek et al., 2014 [4]	Prospective controlled observational cohort	Women aged 12-18 years (68 patients with JIA and 55 healthy)	Bivalent	All participants were seropositive for HPV16/18 one month after the third vaccine dose	Incidence of AEs was comparable between patients and healthy controls. HPV vaccination did not aggravate JIA disease	HPV 16/18-specific antibody concentrations were lower in JIA patients. No effect of methotrexate on HPV16/18 antibodies was detected.

Esposito et al., 2014 [5]	Case-control	Women aged 12-25 years (21 patients with JIA and 21 healthy controls)	Bivalent	All participants were seropositive for HPV16/18 after the third vaccine dose	Incidence of AEs was comparable between patients and healthy controls. No significant changes in JIA disease activity score.	HPV16 neutralising antibody titres were significantly lower in JIA patients vs controls. No differences were observed in HPV18 antibody titres.

AE: adverse events; HPV: human papillomavirus; SLE: systemic lupus erythematosus; JIA: Juvenile Idiopathic Arthritis.

References:

1. Mok CC, Ho LY, Fong LS, To CH. Immunogenicity and safety of a quadrivalent human papillomavirus vaccine in patients with systemic lupus erythematosus: a case-control study. Ann Rheum Dis. 2013;72(5):659-64. http://dx.doi.org/10.1136/annrheumdis-2012-201393 PMID:22589375
2. Heijstek MW, Scherpenisse M, Groot N, Wulffraat NM, Van Der Klis FR. Immunogenicity of the bivalent human papillomavirus vaccine in adolescents with juvenile systemic lupus erythematosus or juvenile dermatomyositis. J Rheumatol. 2013;40(9):1626-7. http://dx.doi.org/10.3899/jrheum.130246 PMID:23997002
3. Soybilgic A, Onel KB, Utset T, Alexander K, Wagner-Weiner L. Safety and immunogenicity of the quadrivalent HPV vaccine in female Systemic Lupus Erythematosus patients aged 12 to 26 years. Pediatr Rheumatol Online J. 2013;11(1):29. http://dx.doi.org/10.1186/1546-0096-11-29 PMID:23924237
4. Heijstek MW, Scherpenisse M, Groot N, Tacke C, Schepp RM, Buisman AM, et al. Immunogenicity and safety of the bivalent HPV vaccine in female patients with juvenile idiopathic arthritis: a prospective controlled observational cohort study. Ann Rheum Dis. 2014;73(8):1500-7. http://dx.doi.org/10.1136/annrheumdis-2013-203429 PMID:23723319
5. Esposito S, Corona F, Barzon L, Cuoco F, Squarzon L, Marcati G, et al. Immunogenicity, safety and tolerability of a bivalent human papillomavirus vaccine in adolescents with juvenile idiopathic arthritis. Expert Rev Vaccines. 2014;13(11):1387-93.

TABLE S8. Published studies investigating the efficacy of quadrivalent human papillomavirus vaccine in patients with recurrent respiratory papillomatosis, January 2006 to June 2016 (n = 9)

Citation	Study design	Population (n)	Vaccine	Seroconversion % (HPV genotype)	Safety	Comments
**Juvenile respiratory papillomatosis**						
Förster et al., 2008 [1]	Case report	1 female<br>Age at clinical onset: 15 months<br>Age at vaccination: 2 years	Quadrivalent	NR	NR	Genotype of lesion: 6 and 11<br>Duration of follow-up: 10 months<br>Outcomes:<br>Complete remission (1)
Mudry et al., 2011 [2]	Case report	1 female<br>Age at clinical onset: 2 years<br>Age at vaccination: 5 years	Quadrivalent	NR	NR	Genotype of lesion: 11<br>Duration of follow-up: 17 months<br>Outcomes:<br>Complete remission (1)
Hočevar-Boltežar et al., 2014 [3]	Case series	4 patients: 3 females and 1 males<br>Age at clinical onset: 1–8 years<br>Age at vaccination: 13–25 years[a]	Quadrivalent	NR	No serious local and systemic adverse reactions	Genotype of lesion: 6 (3 female) and 11 (1 male)<br>Duration of follow-up: 43-52months<br>Outcomes:<br>Partial remission (4)

Chirilă et al., 2014 [4]	Case series	12 patients<br>Age of clinical onset: 2-16 years<br>Age at vaccination: NR	Quadrivalent	NR	23%, 95% CI (8.28–53.25) presented side effects, namely: one patient had a fever (38 °C), one patient had local redness and one had labial herpes[b]	Genotype of lesions: 6 (10 cases) and 11 (3 cases) [b]<br>Duration of follow-up: 12 months<br>Outcomes:<br>Reduction in the number of recurrences with vaccination than with cidofovir<br>[p < 0.001]
Yi et al., 2014 [5]	Case report	1 male patient<br>Age at clinical onset: 2 years<br>Age at vaccination: 6 years	Quadrivalent	NR	NR	Genotype of lesions: 6<br>Duration of follow-up: 6 months<br>Outcomes:<br>Complete remission (1)
Mészner et al., 2015 [6]	Case report	1 male patient<br>Age at clinical onset: 3 months<br>Age at vaccination: 2 years[a]	Quadrivalent	NR	NR	Genotype of lesions: 6 and 11<br>Duration of follow-up: 24 months<br>Outcomes:<br>Complete remission (1)
Young et al., 2015 [7]	Case series	2 male patients<br>Age at clinical onset: 9-16 years[a]<br>Age at vaccination: not reported[a]	Quadrivalent	NR	NR	Outcomes:<br>Complete remission (1)<br>No response (1)

Hermann et al., 2016 [8]	Case series	9 patients: 3 females and 6 males<br>Age at clinical onset:: 1-8 years<br>Age of vaccination: 9-17 years	Quadrivalent	Not reported	None of the patients experienced adverse events	Genotype of lesions: 6 (3 female and 5 male) and 11 (1 male)<br>Duration of follow-up: 12 months<br>Outcomes:<br>No differences pre-post vaccination with regard to clinical score [p = 0.083]; anatomical score [p = 0.257]; interval between surgeries [p = 0.357]; number of surgeries [p = 0.180] and recurrence intervals [p = 0.062]
TOTAL						**Complete remission 5/10 (50%)**<br>**Partial remission 4/10 (40%)**<br>**No response 1/10 (10%)**
Adult respiratory papillomatosis						
Pawlita et al., 2009 [9]	Case report	1 male patient<br>Age at clinical onset: 66 years<br>Age at vaccination: 68 year	Quadrivalent	NR	NR	Genotype of lesions: 6<br>Duration of follow-up: 7 months<br>Outcomes:<br>Complete remission

Hočevar-Boltežar et al., 2014 [3]	Case series	7 patients: 3 females and 4 males<br>Age at clinical onset: 23-44 years<br>Age at vaccination: 26-46 years [a]	Quadrivalent	NR	No serious local or systemic adverse reactions	Genotype of lesions: 6 (4 cases) and 11 (3 cases)<br>Duration of follow-up: 48-52 months<br>Outcomes:<br>Complete remission (1)<br>Partial remission (3)<br>No response (3)
Chirilă et al., 2014 [4]	Case series	18 patients<br>Age at clinical onset: 18-43 years<br>Age at vaccination: not reported	Quadrivalent	NR	23%, 95% CI [8.28–53.25] presented side effects, namely: one patient had a fever (38 °C), one patient had local redness and another one had labial herpes[b]	Genotype of lesions: 6 (10 cases) and 11 (3 cases) [b]<br>Duration of follow-up: 12 months<br>Outcomes:<br>Reduction in the number of recurrences of vaccination compared with cidofovir [p = 0.007]
Young et al., 2015 [7]	Case series	18 patients:<br>8 females and 10 males<br>Age at clinical onset: 27–77 years[a]<br>Age at vaccination: 27–77[a]	Quadrivalent	NR	NR	Genotype of lesions: not reported<br>Duration of follow-up: not reported<br>Outcomes:<br>Complete remission (6)<br>Partial remission (6)<br>No response (6)

**TOTAL**	**Complete remission 8/26 (30%)**<br>**Partial remission 9/26 (35%)**<br>**No response 9/26 (35%)**

CI: confidence interval; F: female; M: male; NR: not reported.

[a]Age at study inclusion.

[b]Total (adult + juvenile).

References:

1. Förster G, Boltze C, Seidel J, Pawlita M, Müller A. Juvenile Larynxpapillomatose – Impfung mit dem polyvalenten Spaltimpfstoff Gardasil.[Juvenile laryngeal papillomatosis--immunisation with the polyvalent vaccine gardasil]. Laryngorhinootologie. 2008;87(11):796-9. German. PMID:18759217
2. Mudry P, Vavrina M, Mazanek P, Machalova M, Litzman J, Sterba J. Recurrent laryngeal papillomatosis: successful treatment with human papillomavirus vaccination. Arch Dis Child. 2011;96(5):476-7. http://dx.doi.org/10.1136/adc.2010.198184 PMID:21220258
3. Hočevar-Boltežar I, Matičič M, Sereg-Bahar M, Gale N, Poljak M, Kocjan B, et al. Human papilloma virus vaccination in patients with an aggressive course of recurrent respiratory papillomatosis. Eur Arch Otorhinolaryngol. 2014;271(12):3255-62. http://dx.doi.org/10.1007/s00405-014-3143-y PMID:24964770
4. . Chirilă M, Bolboacă SD. Clinical efficiency of quadrivalent HPV (types 6/11/16/18) vaccine in patients with recurrent respiratory papillomatosis. Eur Arch Otorhinolaryngol. 2014;271(5):1135-42. http://dx.doi.org/10.1007/s00405-013-2755-y PMID:24121781
5. Yi L, Vaudaux B, Sandu K, Nisa L. Prolonged remission of juvenile-onset respiratory papillomatosis: a post-expositional role of the tetravalent anti-HPV vaccine? Int J Pediatr Otorhinolaryngol. 2014;78(2):388-90. http://dx.doi.org/10.1016/j.ijporl.2013.12.013 PMID:24388316
6. Mészner Z, Jankovics I, Nagy A, Gerlinger I, Katona G. Recurrent laryngeal papillomatosis with oesophageal involvement in a 2 year old boy: successful treatment with the quadrivalent human papillomatosis vaccine. Int J Pediatr Otorhinolaryngol. 2015;79(2):262-6. http://dx.doi.org/10.1016/j.ijporl.2014.11.022 PMID:25496821
7. Young DL, Moore MM, Halstead LA. The use of the quadrivalent human papillomavirus vaccine (gardasil) as adjuvant therapy in the treatment of recurrent respiratory papilloma. J Voice. 2015;29(2):223-9. http://dx.doi.org/10.1016/j.jvoice.2014.08.003 PMID:25619468

8. Hermann JS, Weckx LY, Monteiro Nürmberger J, Santos Junior GF, Campos Pignatari AC, Nagata Pignatari SS. Effectiveness of the human papillomavirus (types 6, 11, 16, and 18) vaccine in the treatment of children with recurrent respiratory papillomatosis. Int J Pediatr Otorhinolaryngol. 2016;83:94-8. http://dx.doi.org/10.1016/j.ijporl.2016.01.032 PMID:26968061

9. Pawlita M, Gissmann L. Rekurrierende respiratorische Papillomatose [Recurrent respiratory papillomatosis]. Dtsch med Wochenschr. 2009;134(Suppl 2):S100-2. German.

Annex. PubMed Search strategy

PubMed Search **User Query - Final:** (HIV OR “Men Who Have Sex With Men” OR MSM OR “Inflammatory Bowel Diseases” OR IBD OR Conization OR “Congenital Bone Marrow Failure Syndrome” OR CBMFS OR immunodeficien* OR “Hematopoietic Stem Cell Transplantation” OR HSCT OR “Bone Marrow Transplantation” OR BMT OR Transplant* OR “Transplant Recipients” OR autoimmun* OR biologic OR anti-TNF OR immunocompromi* OR immunosuppress* OR chemotherap* OR radiation OR steroid* OR “Recurrent respiratory papillomatosis” OR RRP) AND (“Papillomavirus Vaccines” OR Cervarix OR Gardasil OR ((“Human papillomavirus” OR HPV) AND (vaccin* OR immunis* OR immuniz*))) AND (effective* OR efficac* OR immunogenic* OR safe*) AND ((“2006/01/01”[PDat]: “2016/06/30”[PDat]) AND Humans[Mesh] AND (English[lang] OR Spanish[lang]))</unknown>
